# Active Component of *Antrodia cinnamomea* Mycelia Targeting Head and Neck Cancer Initiating Cells through Exaggerated Autophagic Cell Death

**DOI:** 10.1155/2013/946451

**Published:** 2013-06-12

**Authors:** Ching-Wen Chang, Chien-Chih Chen, Meng-Ju Wu, Yu-Syuan Chen, Chin-Chu Chen, Sen-Je Sheu, Ting-Wei Lin, Shiu-Huey Chou, Shu-Chun Lin, Chung-Ji Liu, Te-Chang Lee, Chih-Yang Huang, Jeng-Fan Lo

**Affiliations:** ^1^Institute of Oral Biology, National Yang-Ming University, No. 155, Section 2, Li-Nong Street, Taipei 11221, Taiwan; ^2^Department of Biotechnology, Hungkuang University, Taichung, Taiwan; ^3^Grape King Inc., Taoyuan County, Taiwan; ^4^Department of Life Science, Fu-Jen University, New Taipei City, Taiwan; ^5^Department of Oral and Maxillofacial Surgery, Mackay Memorial Hospital, Taipei, Taiwan; ^6^Institute of Biomedical Sciences, Academia Sinica, Taipei, Taiwan; ^7^Graduate Institute of Chinese Medical Science and Institute of Medical Science, China Medical University, No. 91, Hsueh-Shih Road, Taichung 40402, Taiwan; ^8^Institute of Basic Medical Science, China Medical University, Taichung, Taiwan; ^9^Department of Health and Nutrition Biotechnology, Asia University, Taichung, Taiwan; ^10^Cancer Research Center & Genome Research Center, National Yang-Ming University, Taipei, Taiwan; ^11^Department of Dentistry, Taipei Veterans General Hospital, Taipei, Taiwan; ^12^National Yang-Ming University, VGH Genome Research Center, Taipei, Taiwan

## Abstract

Head and neck squamous cell carcinoma (HNSCC) is a highly lethal cancer. Previously, we identify head and neck cancer initiating cells (HN-CICs), which are highly tumorigenic and resistant to conventional therapy. Therefore, development of drug candidates that effectively target HN-CICs would benefit future head and neck cancer therapy. In this study, we first successfully screened for an active component, named YMGKI-1, from natural products of *Antrodia cinnamomea* Mycelia (ACM), which can target the stemness properties of HNSCC. Treatment of YMGKI-1 significantly downregulated the aldehyde dehydrogenase (ALDH) activity, one of the characteristics of CIC in HNSCC cells. Additionally, the tumorigenic properties of HNSCC cells were attenuated by YMGKI-1 treatment *in vivo*. Further, the stemness properties of HN-CICs, which are responsible for the malignancy of HNSCC, were also diminished by YMGKI-1 treatment. Strikingly, YMGKI-1 also effectively suppressed the cell viability of HN-CICs but not normal stem cells. Finally, YMGKI-1 induces the cell death of HN-CICs by dysregulating the exaggerated autophagic signaling pathways. Together, our results indicate that YMGKI-1 successfully lessens stemness properties and tumorigenicity of HN-CICs. These findings provide a new drug candidate from purified components of ACM as an alternative therapy for head and neck cancer in the future.

## 1. Introduction

Head and neck squamous cell carcinoma (HNSCC) represents the sixth most common cancer worldwide and the third most common cancer in developing nations [1]. Despite the recent advancements in the multidisciplinary treatment of HNSCC, prognosis of patients with locally advanced diseases and long-term survival rates remains unsatisfactory [2]. Over the past decade, increasing evidence suggests that the hierarchical model of cancer initiating cells (CICs) or cancer stem cells (CSCs) in that each tumor formation is governed by a rare subpopulation of cells with self-renewal capacity [3]. CICs have been demonstrated to have capacities of promoting tumor growth, tumor regeneration, metastatic progression, and tumor recurrence [4, 5]. It is imperative to uncover new therapeutic drug on targeting cancer stem cells [6].

 Previously, we have verified a subpopulation of HNSCC cells (HNSCCs) displaying the characteristics of CICs [5]. In addition, we have identified molecular markers such as CD133 [8] and ^mem^GRP78 (membrane anchoring GRP78) [9] for targeting CICs. Others have found that Aldehyde dehydrogenase (ALDH), which has been demonstrated as a breast cancer CIC marker [10], could also be the CIC marker of head and neck cancer and other cancers [11, 12]. More recently, we have successfully verified an epithelial-mesenchymal transition (EMT) related gene expression, S100A4, which is upregulated in HN-CICs. Consequently, we have shown that significant downregulation of ALDH activity comes along with loss of stemness properties in S100A4 knockdown HNSCCs and HN-CICs [13]. Therefore, the cell-based ALDH activity assay is useful as a screening system for drug candidates, which would target the stemness properties of cells. With the assay system, we would be able to screen for active components of natural products from Chinese medicinal herbals on target cellular ALDH activity *in vitro*.

It has been reported that CICs, bearing highly malignant stem cell traits acquired through processes such as the EMT, would survive during metabolic stress only if they adopt adaptive mechanisms such as autophagy [14]. However, autophagy has a dual role in CICs [15]. Autophagy has been linked to the maintenance of breast cancer stem-like phenotype [16]. In contrast, some studies demonstrate that autophagy plays an essential role in differentiation of glioma-initiating cells [17] and in inhibition of colon cancer stem cell [18]. Thus, the role of autophagy in CICs remains elusive.

*Antrodia cinnamomea*, a rare mushroom of the family Polyporaceae, only grows naturally in Taiwan [19]. For Taiwanese traditional medicinal treatment, *Antrodia cinnamomea* has been applied for diarrhea, intoxication, hypertension, abdominal pain, itchy skin [20], and cancers therapy [21]. The biological or physiological functions of the crude extracts or purified active components of fruiting bodies or submerged cultured mycelia of *Antrodia cinnamomea* have been examined. Empirically, these active components of natural products from *Antrodia cinnamomea* showed antioxidant [20], anti-inflammatory [22], hepatoprotective [23], and antitumor activities. Further, the antitumor activities of *Antrodia cinnamomea* have recently become popular as an alternative therapeutic agent for several types of human cancer [21, 24]. 

Several pure compounds in the fruiting body of *Antrodia cinnamomea* have been isolated and identified with antitumor activity [25]. Compounds of maleic and succinic acid derivatives from the mycelium of *Antrodia cinnamomea* exhibit significant cytotoxic effects on Lewis lung carcinoma (LLC) tumor cell [26]. However, the inhibitory effect of *Antrodia cinnamomea* on the cancer initiating cells has never been studied. Therefore, we are interested in screening and evaluating the effects of active components from *Antrodia cinnamomea* on targeting CICs and in elucidating the possible biological mechanisms mediating the antitumor activity *in vitro* and *in vivo*.

Herein, we exploited the perivous *in vitro* cell-based ALDH activity assay system to screen for the active components from *Antrodia cinnamomea* Mycelia extracts (ACMEs) with effective cytotoxicity toward to CICs. We used both *in vitro* assays and *in vivo* xenograft mouse models to examine the cytotoxic effects of a purified component of ACME, YMGKI-1 (a derivative of maleic and succinic acid), on the HN-CICs. Strikingly, YMGKI-1 treatment diminished stemness properties and tumorigenicity of HN-CICs both *in vitro* and *in vivo*. Finally, we demonstrated that the induction of cell death in YMGKI-1-treated HN-CICs is through dysregulatory autophagic signaling, which is regulated mainly by mTOR molecular pathway. Thus, our study indicates that we have successfully isolated active compounds from natural products of *Antrodia cinnamomea* Mycelia (ACM) with selective and effective cytotoxicity on HN-CICs. Further, our findings provide a new drug candidate as an alternative therapy for head and neck cancer in the future.

## 2. Materials and Methods

### 2.1. Extraction, Isolation, Purification, and Structure Determination of Single Compounds from the ACM

YMGKI-1 (3-[4-(3-Methylbut-2-enyloxy)phenyl]-4-isobutyl-N-hydroxypyrrole-2,5-dione; Mw: 329 Da) from ACMs and the chemical structure of the purified chemicals were performed and determined by Dr. Chien-Chih Chen (Hungkuang University, Taichung, Taiwan). The purification procedure of YMGKI-I from ACMs was described in Supplementary Materials and Methods available online at http://dx.doi.org/10.1155/2013/946451. Dimethyl sulfoxide (DMSO) (Sigma-Aldrich D2650, Saint Louis, MO) is used as a drug solvent.

### 2.2. Cell Lines

Human tongue carcinoma cells (SAS) were obtained from the Japanese Collection of Research Bioresources (Tokyo, Japan) [27]. Human gingival squamous carcinoma cells (OECM-1) were provided from Dr. C. L. Meng (National Defense Medical College, Taipei, Taiwan). Primary culture of normal human oral keratinocytes (NHOKs) was as described [5]. Additionally, the amniotic fluid stem cells-2 (AFSC-2) and hematopoietic stem cell (HSC) were isolated by Dr. Shiu-Huey Chou at Fu-Jen University. The isolation protocols of AFSC-2 and HSC were described in Supplementary Materials and Methods.

### 2.3. Antibodies and Reagents

Anti-Oct-4 (MAB430), anti-Nanog (AB9220), anti-CD133 (MAB4310), and anti-GAPDH (MAB374) were obtained from Millipore Corporation (Billerica, MA). Anti-GRP78 (610978) and anti-E-cadherin (610182) were obtained from BD Bioscience (San Jose, CA). Anti-Notch2 (sc-5545) and anti-PI3K (sc-1637) were obtained from Santa Cruz Biotechnology, Inc. (Santa Cruz, CA). Anti-LC3 (no. 81631), anti-phosphor mTOR (Ser2448) (no. 2971), anti-phosphor p44/p42 MAPK (Thr202/Tyr204) (no. 9101), anti-phosphor p38 MAPK (Thr180/Tyr182) (no. 9211), anti-HER2 (no. 2165), and anti-phosphor EGFR (Tyr1068) (no. 2234) were obtained from Cell Signaling Technology, Inc. (Danvers, MA). 3-Methyladenine (3-MA) (M9281), Metformin (Mf) (D150959) was obtained from Sigma-Aldrich (Saint Louis, MO).

### 2.4. Cell Lines Cultivation and Enrichment of HN-CICs

In brief, SAS and OECM1 were grown in DMEM or in RPMI supplemented with 10% FBS (Grand Island, NY), respectively. The SAS cells were then cultured in tumor sphere medium consisting of serum-free DMEM/F12 medium (GIBCO), N2 supplement (GIBCO), 10 ng/mL human recombinant basic fibroblast growth factor-basic (FGF), and 10 ng/mL Epidermal Growth Factor (EGF) (R&D Systems, Minneapolis, MN). Cells were plated at a density of 7.5 × 10^4^ live cells/10 mm dish, and the medium was changed every other day until the tumor sphere formation was achieved to enrich the SAS-HN-CICs or OECM1-HN-CICs in about 4 weeks [5]. 

### 2.5. ALDH Activity Assay

The ALDEFLUOR kit (Stem Cell Technologies, Durham, NC, USA) was used to examine the ALDH enzymatic activity [13]. Single cell suspension obtained from cancer cells with or without treatment was suspended in ALDEFLUOR assay buffer containing ALDH substrate (BAAA, 1 *μ*mol/L per 1 × 10^6^ cells) and incubated during 40 minutes at 37°C. As negative control, for each sample of cells an aliquot was treated with 50 mmol/L diethylaminobenzaldehyde (DEAB), a specific ALDH inhibitor. This resulted in a significant decrease in the fluorescence intensity of ALDH-positive cells and was used to identify the ALDH-positive cells. The amount of intracellular fluorescence was measured by FACS Calibur apparatus (Becton Dickinson, San Diego, CA).

### 2.6. Side Population Analysis

Cells were resuspended at 1 × 10^6^/mL in prewarmed DMEM with 2% FCS. Hoechst 33342 dye was added at a final concentration of 5 *μ*g/mL in the presence or absence of fumitremorgin C (FTC) (10 *μ*M; Sigma, St Louis, MO, USA) and was incubated at 37°C for 90 min with intermittent shaking. At the end of the incubation, the cells were washed with ice-cold HBSS with 2% FCS and centrifuged down at 4°C and resuspended in ice-cold HBSS containing 2% FCS. Propidium iodide at a final concentration of 2 *μ*g/mL was added to the cells to gate viable cells. The cells were filtered through a 40 *μ*m cell strainer to obtain single cell suspension before analysis. The Hoechst 33342 dye was excited at 357 nm and its fluorescence was dual-wavelength analyzed (blue, 402–446 nm; red, 650–670 nm). Analyses were done on FACS Aria (Becton Dickinson, San Diego, CA).

### 2.7. Cell Viability Assay

Cell viability was measured by MTT assay based on the ability of live cells to convert tetrazolium salt into purple formazan. Cells were seeded onto 24-well dishes with medium containing YMGKI-1 at concentrations ranging from 0 to 35 *μ*g/mL for 24 hr. After treatment for the indicated times, 5 *μ*L of MTT solution (4 mg MTT/mL PBS) was added to each well and the plate was incubated at 37°C for 3 h. After the removal of medium, 100 *μ*L of DMSO was added to each well and the plate was gently shaken for 30 min. The absorbance was determined at 560 nm. The cell viability was calculated as OD of experimental groups/OD of control groups × 100%.

### 2.8. Apoptotic Assay

Apoptotic cells were detected with an Annexin V-APC kit (Calbiochem, Darmstadt, Germany) according to manufacturer's guidelines. After staining, the cells were incubated with 20 *μ*g/mL propidium iodide (PI) to gate the dead cells. Consequently, the fluorescent signals from Annexin V or PI were analyzed by FACS Canto apparatus (Becton Dickinson, San Diego, CA, USA).

### 2.9. Matrigel Invasion Assay

For transwell migration assays, 2 × 10^5^ cells were plated into the top chamber of a transwell (Corning, Acton, MA) with a porous transparent polyethylene terephthalate membrane (8.0 *μ*m pore size). Cells were plated in medium with lower serum (0.5% FBS), and medium supplemented with higher serum (10% FBS) was used as a chemoattractant in the lower chamber. The cells were incubated for 24 h and cells that did not migrate through the pores were removed by a cotton swab. Cells on the lower surface of the membrane were stained with Hoechst 33258 (Sigma-Aldrich) to show the nuclei; fluorescence was detected at a magnification of 100x using a fluorescence microscope (Carl Zeiss, Oberkochen, Germany). The number of fluorescent cells in a total of five randomly selected fields was counted.

### 2.10. Anchorage Independent Growth Assay

Each well (35 mm) of a six-well culture dish was coated with 2 mL bottom agar (Sigma-Aldrich) mixture (DMEM, 10% (v/v) FBS, 0.6% (w/v) agar). After the bottom layer was solidified, 2 mL top agar-medium mixture (DMEM, 10% (v/v) FBS, 0.3% (w/v) agar) containing 2 × 10^4^ cells was added, and the dishes were incubated at 37°C for 2 weeks. Plates were stained with 0.005% Crystal Violet and then the colonies were counted. The number of total colonies was counted over five fields per well for a total of 15 fields in triplicate experiments.

### 2.11. Acridine Orange (AO) Staining Assay

Cells were washed with Hank's buffered salt solution (HBSS) twice, followed by staining with 1 *μ*g/mL acridine orange (Sigma, A 6014), and diluted in HBSS containing 5% FBS for 15 min at 37°C. After staining, cells were washed ice-cold HBSS with 5% FBS and centrifuged down at 4°C and resuspended in ice-cold HBSS containing 5% FBS. The stained cells were observed under a red filter fluorescence microscope. To quantify the AVO formation, the acridine orange stained cells were resuspended in HBSS containing 5% FBS and analyzed by FACS Canto apparatus (Becton Dickinson, San Diego, CA, USA) [28].

### 2.12. *In Vivo* Tumorigenic Assay

All the animal practices in this study were approved and in accordance with the Institutional Animal Care and Use Committee (IACUC) of National Yang-Ming University, Taipei, Taiwan (IACUC approval nos. 1001223 and 991235). The effect of YMGKI-1 on anti-tumorigenic activity was examined in 8-week-old nude BALB/c nu/nu mice (*n* = 3 per group). SAS cells exposed to YMGKI-1 (1 × 10^6^) were subcutaneously injected into the subcutaneous of nude mice. Tumors were generally palpable at 5 to 7 days after inoculation and tumor volumes were measured (using a caliper and calculated as length × width^2^  × 0.5) twice weekly until 28 days after injection. To determine the therapeutic effect of YMGKI-1 on regression, SAS cells (5 × 10^5^ cells in 0.1 mL DMEM) were inoculated subcutaneously into the back of nude BALB/c nu/nu mice (*n* = 3 per group). Tumors were allowed to develop to be palpable. Then, the YMGKI-1 was administered intraperitoneally at days 11, 13, and 17 after SAS cell inoculation, and tumor volumes were determined twice weekly. The tumor volume was calculated according to the formula: (Length × Width^2^)/2 [5].

### 2.13. Statistics

An unpaired *t*-test was used for the statistical analysis. The results were considered to be statistically different when the *P* value was <0.05.

## 3. Results

### 3.1. Effective Targeting of HN-CICs by YMGKI-1 Treatment

**Figure 1:**
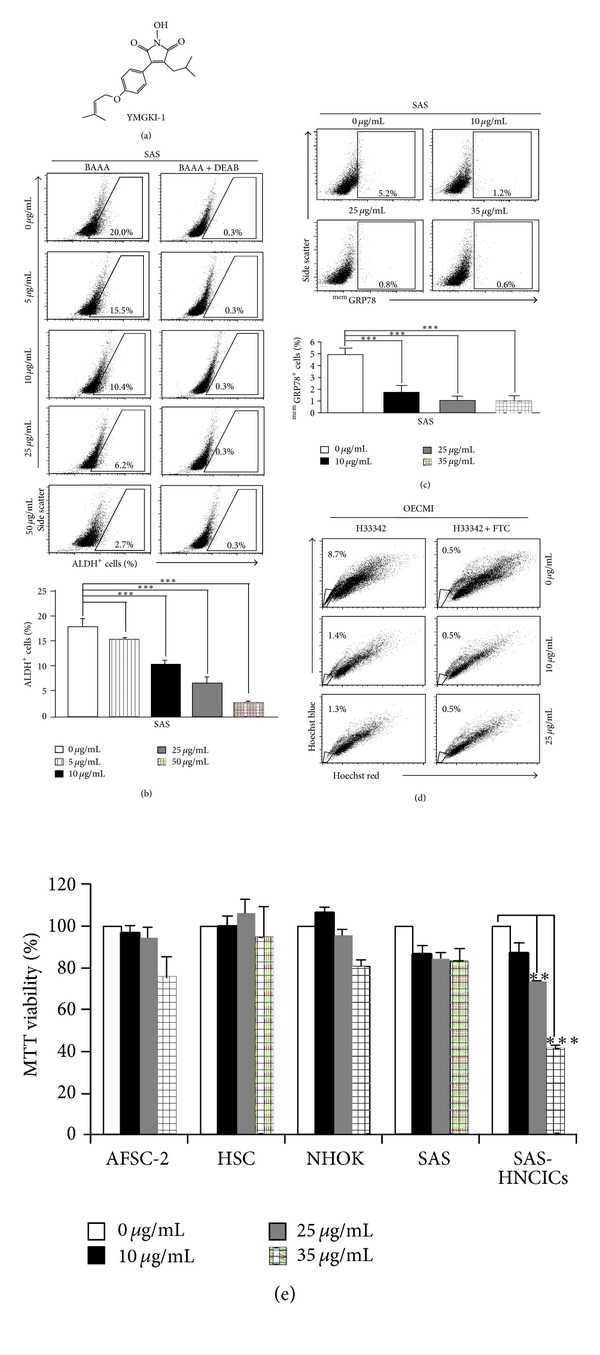
YMGKI-1 treatment effectively affects CIC subpopulation of HNSCC cells but not normal stem cells. (a) Chemical structure of YMGKI-1 isolated from the mycelium of *Antrodia cinnamomea*. (b) SAS cells were treated with 0, 10, 25, and 50 *μ*g/mL of YMGKI-1 for 24 hr, afterward; the ALDH activity of YMGKI-1 treated cells was examined by flow cytometry. DEAB, the inhibitor of ALDH1 enzyme, was used to verify the ALDH-positive cells. The ^mem^GRP78^+^ cells (c) or side population (d) of YMGKI-1-treated SAS or OECM1 cells were stained by anti-GRP78 antibody or Hoechst 33342 (see Section 2), respectively, and analyzed by flow cytometry. (e) The cell viability of YMGKI-1-treated parental SAS cells, normal human oral keratinocytes (NHOKs), amniotic fluid stem cells-2 (AFSC-2), and hematopoietic stem cell (HSC) and SAS-HN-CICs was measured by MTT assay, respectively. The data are means ± SD of triplicate samples from three experiments (**P < 0.01; ***P < 0.001). The same concentration (0.03%) of vehicle (DMSO) was added to all control groups.

Cancer-initiating cells (CICs) are a rare subpopulation of cancer cells, which are responsible for tumor growth and cancer recurrence during conventional chemotherapy or radiotherapy [3, 4]. High ALDH activity has been used as a selection marker to isolated breast cancer CICs or head and neck CICs (HN-CICs) [29]. Therefore, we first utilized the *in vitro* cell-based aldehyde dehydrogenase (ALDH) activity assay system to screen for the active components from *Antrodia cinnamomea* mycelia extract (ACME) with effective reduction on ALDH activity of cells. Of the tested compounds purified from ACME, YMGKI-1 (a malic and succinic acid derivative (Figure 1(a))) significantly reduced the ALDH enzymatic activity of YMGKI-1-treated SAS cells in a dose-dependent manner (Figure 1(b)). We also found that YMGKI-1 diminished the ALDH enzyme activity in other human cancer cell lines (Supplementary Table 1). Next, treatment with YMGKI-1 also reduced the percentage of ^mem^GRP78^+^ cells in HNSCC cells, where ^mem^GRP78 has been used as a cell surface marker for isolation of HN-CICs (Figure 1(c)) [9]. In Figure 1(d), we showed that treatment of YMGKI-1 in OECM1 cells caused the decreasing of the side population (SP) cells, which is also one of the characteristics of CICs [30]. To further evaluate the inhibitory effect of YMGKI-1 treatment on different cell lines including normal human oral keratinocytes (NHOKs), amniotic fluid stem cells-2 (AFSC-2), hematopoietic stem cell (HSC), parental SAS, and SAS-HN-CICs, the MTT cell proliferation assay was applied. Interestingly, we observed a massive decrease in viability of the SAS-HN-CICs but no significant inhibition on the other cells (Figure 1(e); **P < 0.01; ***P < 0.001). Hence, the previous findings suggest that YMGKI-1 might effectively and selectively target HN-CICs.

### 3.2. Reduction of Xenograft Tumor Growth on YMGKI-1-Treated HNSCC Cells

**Figure 2:**
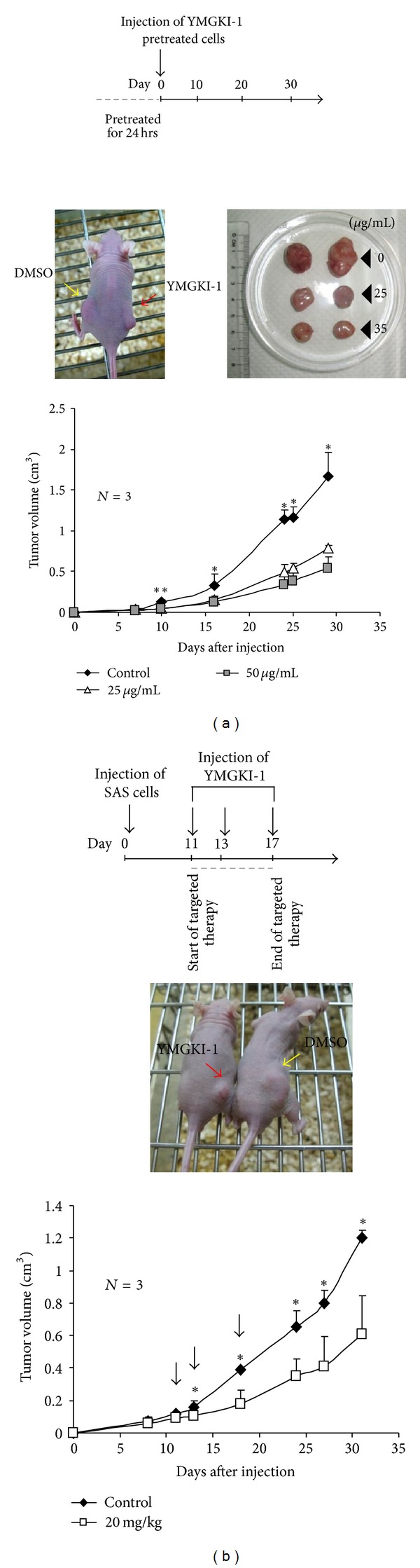
YMGKI-1 treatment suppresses xenograft tumor growth* in vivo*. (a) SAS cells pretreated with YMGKI-1 (0, 25, 50 *μ*g/mL) for 24 hr were injected into the subcutaneous space of nude mice. Representative image of nude mice displaying tumor growth caused by either control SAS cells (DMSO) injected into the left subcutaneous space or SAS cells with pretreatment of YMGKI-1 (25 *μ*g/mL) injected into the right subcutaneous space on day 20 (middle left panel). Image of dissected tumors collected on day 30 (left column: DMSO (control), middle column: treated with 25 *μ*g/mL, and right column: treated with 50 *μ*g/mL) (middle right panel). The tumor growth curves on nude mice inoculated with YMGKI-1 pretreated SAS cells were recorded (lower panel). (b) Therapeutic model to demonstrate the effect of YMGKI-1 treatment on inhibiting tumor growth. Parental SAS cells (1 × 10^6^ cells) were subcutaneously implanted into the right back of nude mice and allowed to develop tumors to a size around 0.1 cm^3^. On days 11, 13, and 17 after cells implantation, nude mice bearing SAS-derived tumors were intraperitoneally injected with YMGKI-1 (20 mg/kg) or DMSO (as control). On day 24, the image of mice, including control mouse (DMSO) on the right side plus experimental mouse (YMGKI-1 treatment) on the left side, was collected (middle panel). Additionally, the tumor growth curves were recorded (bottom panel). Error bars correspond to SD (*n* = 3; *P < 0.05; **P < 0.01).

Existence of HN-CICs is critical for the tumor growth of HNSCC *in vivo* [5, 8, 9, 13]. In Figure 1, we showed that YMGKI-1 treatment effectively diminished the stemness properties of HN-CICs in HNSCCs. Therefore, we wanted to determine if YMGKI-1 treatment could attenuate the tumor initiating activity of HNSCC *in vivo*. As shown in Figure 2(a), nude mice injected with YMGKI-1 pretreated SAS cells, a tumorigenic cell line, displayed significant reduction of the tumor growth as compared to those mice injected with untreated SAS cells (*P < 0.05; **P < 0.01). Next, whether YMGKI-1 could function as a therapeutic reagent on attenuating the tumor growth was examined as follow. We first subcutaneously implanted parental SAS cells into nude mice and allowed the tumors to be established. Mice bearing palpable tumor were then intraperitoneally injected with YMGKI-1 or DMSO (as control). Effectively, tumor-bearing mice receiving YMGKI-1 treatment afterward displayed reduced tumor size and the tumor growth curves in comparison with the control mice (Figure 2(b)) (*P < 0.05; **P < 0.01). In the meantime, we did not observe severe side effect but with little body weight loss from mice with YMGKI-1 treatment (data not shown). 

### 3.3. Diminished Stemness Properties and Enhanced Differentiation Ability in YMGKI-1-Treated HN-CICs

**Figure 3:**
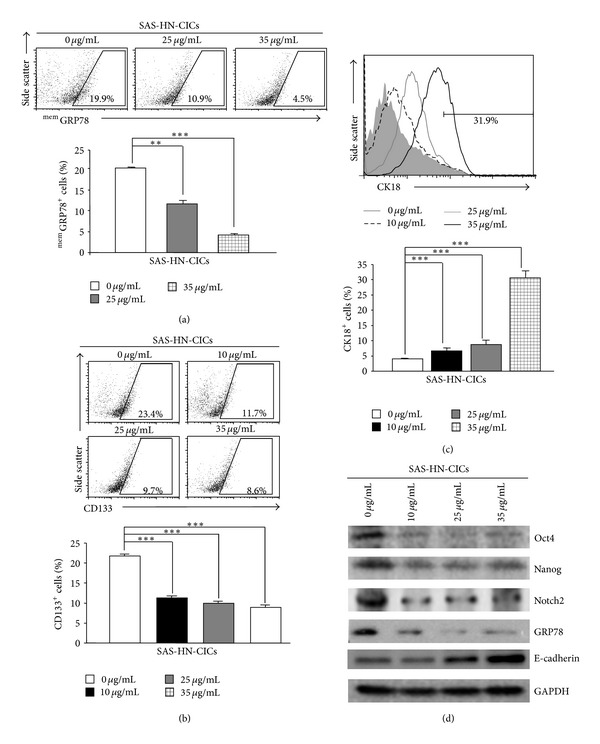
YMGKI-1 treatment diminishes stemness properties and enhances differentiation capability in HN-CICs. SAS-HN-CICs were treated with YMGKI-1 at different concentration for 24 hr, then stained with anti-GRP78 (a), anti-CD133 (b), or anti-CK18 (c) antibodies, respectively, and quantitated by flow cytometry. Data are means ± SD of triplicate samples from three experiments (**P < 0.01; ***P < 0.005). Dead cells were excluded by gating on the propidium-iodide- (PI-) positive cell fraction. (d) Crude cell extract proteins of YMGKI-1-treated-SAS-HN-CICs were collected, electrophores, and analyzed by immunoblotting against anti-Oct-4, anti-Nanog, anti-Notch2, anti-GRP78, anti-E-cadherin, or anti-GAPDH antibodies as indicated. The immunoactive signal of GAPDH protein of different crude cell extracts was referred as loading control. The same concentration (0.03%) of vehicle (DMSO) was added to all control groups.

To further evaluate whether treatment of YMGKI-1 would hinder the stemness properties of HN-CICs, we examined the following stemness properties of YMGKI-1-treated HN-CICs. We first observed that YMGKI-1 treatment significantly decreased the percentage of ^mem^GRP78^+^ or CD133^+^ cells in enriched SAS-HN-CICs (Figures 3(a) and 3(b)), where ^mem^GRP78^+^ or CD133^+^ cells have been demonstrated as CICs with higher stemness properties [5, 9]. Inversely, SAS-HN-CICs with the YMGKI-1 treatment displayed enhanced expression of epithelial differentiation marker, cytokeratin 18 (CK-18) (Figure 3(c)). Immunoblot analyses also showed that the expression of “cancer stemness” proteins (Oct-4, Nanog and Notch2) and GRP78 was diminished in YMGKI-1-treated SAS-HN-CICs (Figure 3(d)). In contrast, the YMGKI-1-treated SAS-HN-CICs displayed increased expression of epithelial-like protein (E-Cadherin) (Figure 3(d)). Together, YMGKI-1 treatment diminished the stemness properties but enhanced the differentiation ability of HN-CICs.

### 3.4. Reduction of Cell Survival and Malignancy of HN-CICs by YMGKI-1 Treatment *In Vitro*

Herein, we wanted to determine whether YMGKI-1 treatment affects cell survival and malignancy of HN-CICs or HNSCCs *in vitro*. Cells were first treated with YMGKI-1 and analyzed by flow cytometry, and the cells stained positively with propidium iodide (PI) were accounted as “dead” cells. Interestingly, YMGKI-1 treatment significantly induced cell death in SAS-HN-CICs whereas no significant cytotoxicity or morphological change was observed in YMGKI-1-treated parental SAS cells (Supplementary Figures S1A and S1B). Collectively, these results suggest that YMGKI-1 effectively suppresses the growth of SAS-HN-CICs but not parental SAS cells. Further, the *in vitro* malignancy of YMGKI-1-treated SAS-HN-CICs including matrigel invasion ability and anchorage independent growth was evaluated. As shown in Figures 3(b) and 3(c), the invasiveness and soft agar colony formation abilities of YMGKI-1 treated SAS-HN-CICs were significantly reduced. 

### 3.5. Induction of Autophagic Cell Death in YMGKI-1-Treated HN-CICs

Due to the observation of induced cell death in YMGKI-1-treated HN-CICs (Supplementary Figures S1A and S1B), next, we wanted to examine whether the dying cells of YMGKI-1-treated HN-CICs were undergoing apoptosis. Apoptosis is designated as type I programmed cell death, and the apoptotic cells are determined by costaining with Annexin-V (AV) and propidium iodide (PI) [31]. When SAS-HN-CICs were cultivated with YMGKI-1 of 35 *μ*g/mL, there was a slight decrease in early apoptotic cells (AV^+^/PI^−^) (Figure 5(a); untreated: 2.1% versus treated: 1.8%) but a significant increase in later apoptotic cell death (AV^+^/PI^+^) (Figure 5(a); untreated: 3.7% versus treated: 23.6%). Nevertheless, many YMGKI-1 treated cells could die from the other programmed cell death such as autophagy (untreated: (AV^−^/PI^+^: 6.7%) versus treated: (AV^−^/PI^+^: 40.8%)) (Figure 5(a)). 

To further understand the molecular mechanism by which to cause the YMGKI-1 induced cell death in HN-CICs, we wanted to determine whether YMGKI-1 treatment induced autophagy in HN-CICs. Acidification of autolysosomes caused by lysosomes fusion with autophagosomes is regarded as an indication of cell autophagy, and autophagy is characterized by AVO formation, which can be detected and measured by acridine orange staining [32]. Using acridine orange staining, we showed progressive increasing in formation of AVO in a dose-dependent manner of YMGKI-1 treated HN-CICs (Figure 5(b)). Further, under the treatment with 10, 25, or 35 *μ*g/mL concentrations of YMGKI-1, we detected that around 12%, 20%, or 43% of HN-CICs underwent autophagy by flow cytometry analyses, respectively (Figure 5(c)).

Autophagy is defined as type II programmed cell death and could be also determined by the induction of LC3-II [33]. Thus, we wanted to utilize the amount of LC3-II or the LC3-II/LC3-I ratio, which is positively correlated with the number of autophagosomes, to identify whether the YMGKI-1-induced dying cells were undergoing autophagy. As shown in Figure 5(d), an increase of LC3-II/LC3-I ratio in YMGKI-1 treated cells was observed in a dose-dependent manner. To further confirm that autophagic cell death was responsible for the cell death of YMGKI-1-treated HN-CICs, we evaluated the protective effect of 3-methyladenine (3-MA; a prototypic autophagy inhibitor [33]) in HN-CICs with combined treatment with YMGKI-1 and 3-MA. As expected, 3-MA cotreatment attenuated the cell death and the LC3-II accumulation induced by YMGKI-1 treatment in HN-CICs (Figures 5(e) and 5(f)). 

We also examined the effects of YMGKI-1 treatment on the expression of apoptosis-related proteins in HN-CICs (Supplementary Figure S2A). HN-CICs treated with YMGKI-1 resulted in cleavage of caspase-3 and poly (ADP-ribose) polymerase (PARP) at 35 *μ*g/mL but not at 10 or 25 *μ*g/mL. Recent data support that the execution of apoptosis could be dependent on the occurrence of autophagy [33, 34]. In summary, our results suggest that YMGKI-1-treated HN-CICs were undergoing the activation of autophagic cell death by which to cause apoptosis.

### 3.6. Affected Signaling Pathways in YMGKI-1-Treated HN-CICs

The activation of mTOR-mediated signaling pathway is known to inhibit autophagy. In addition, the mTOR pathway is activated for self-renewal, cell survival, and malignancy of CICs [35]. In addition, HER2 overexpression is reported to be able to increase the stem/progenitor cell population in malignant mammary cells. Increased levels of HER2 can activate the PI3K/Akt pathway which is related to self-renewal ability in stem cells [36]. Further, activation of phosphor-AMPK can inactivate the mTOR complex-1, inversely, to cause the activation of autophagy [37].

Herein, we examined the effect of YMGKI-1 on these signaling molecules by immunoblot analyses. As shown in Figures 6(a) and 6(b), YMGKI-1 treatment in HN-CICs effectively decreased the expression level of phosphor-mTOR, HER2, phosphor-EGFR, Phosphatidylinositol 3-kinases (PI3K), phosphor-p44/42 MAPK (Thr202/Tyr204), and phosphor-AMPK but not phosphor-p38 MAPK in a dose-dependent manner. Additionally, cotreatment of 3-MA attenuated the expression of phospho-AMPK, which is induced by YMGKI-1 treatment in HN-CICs (Figure 6(b)). It has been reported that AMPK activator like metformin or EGCG selectively kills cancer stem cells [38, 39]. To address the role of AMPK and mTOR on mediating HN-CICs through autophagy, we treated HN-CICs with AMPK activators metformin (Mf) or mTOR inhibitors rapamycin (Rapa), respectively. As shown in Figure 6(c), HN-CICs treated with metformin or rapamycin displayed slight induction of LC3-II along with upregulation of phosphor-AMPK or downregulation of phosphor-mTOR, respectively (Figure 6(c)). In the meantime, we did not observe significant cell death from HN-CICs under metformin or rapamycin treatment (Supplementary Figure S3B). These data suggest that YMGKI-1 treatment in HN-CICs simultaneously induced dysregulatory autophagic cell death through multiple mechanisms.

### 3.7. Attenuation of Inhibitory Effect of YMGKI-1 by Autophagy Repressor on Sphere Formation Ability of HN-CICs

To further investigate whether the “antistemness” properties of YMGKI-1 are autophagy dependent, the sphere formation ability of YMGKI-1-treated HN-CICs was analyzed. As shown in Figure 6(d), YMGKI-1 treatment significantly inhibited the sphere formation ability of HN-CICs. Further, cotreatment of 3-MA lessened the inhibitory effect of YMGKI-1 on sphere formation ability. The results indicated that YMGKI-1 treatment inhibited the stemness properties through autophagy activation in HN-CICs.

### 3.8. Autophagic Pathway and Putative Molecules Regulated by YMGKI-1

It has been known that cells undergoing epithelial-mesenchymal transition (EMT) can gain stem cell properties [40]. We also observed that YMGKI-1 treatment not only increased the expression of CK-18, an epithelial differentiation marker, but also reduced the expression of “cancer stemness” genes and markers in HN-CICs (Figures 1(b)–1(d), Figures 3(a)–3(d)). Intriguingly, Tseng et al. predict that Himanimide-C, a YMGKI-1 analogue, might be a potential inhibitor of cyclooxygenase-2 (COX-2) and 5-lipoxygenase (5-LOX) [7], which play important roles in EMT [40, 41]. Collectively, we hypothesize that YMGKI-1 might suppress EMT and stemness properties of HN-CICs by inhibiting 5-LOX or COX-2. Therefore, we proposed a molecular signaling pathway in HN-CICs responding to YMGKI-1 treatment (Figure 7). Overall, YMGKI-1 would directly or indirectly regulate the autophagic pathway mediated by mTOR and AMPK, in addition to the aforementioned putative COX-2 or 5-LOX molecules, by which YMGKI-1 regulates the stemness, tumorigenicity, differentiation ability, and cell death of HN-CICs. The future research to delineate the function of YMGKI-1 or YMGKI-1 derivatives on HN-CICs will benefit future cancer therapeutics. 

## 4. Discussion 

**Figure 4:**
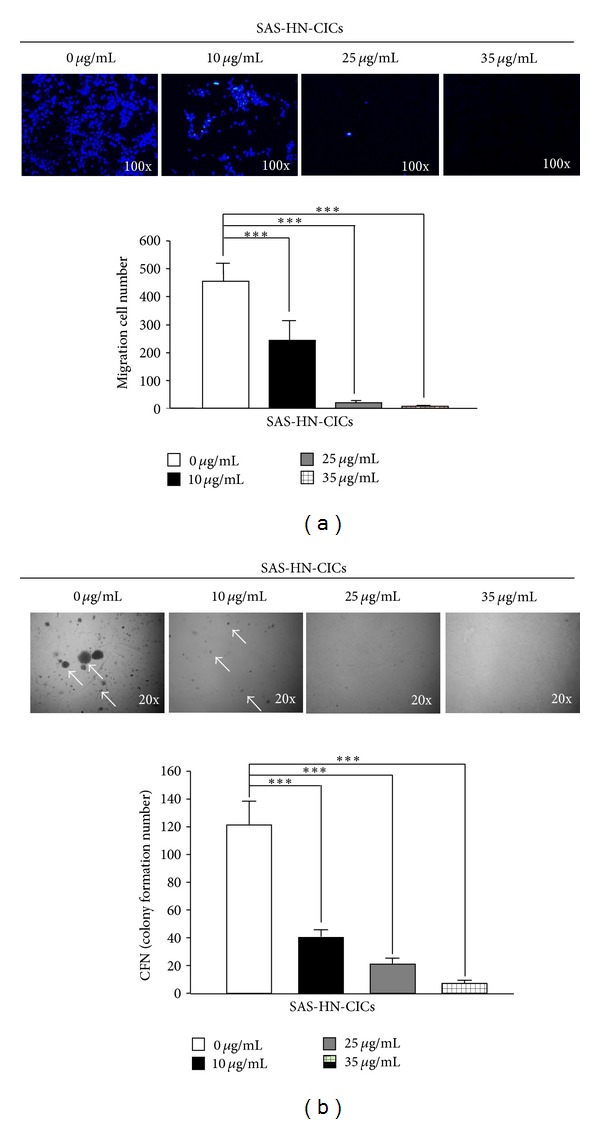
YMGKI-1 treatment reduces cell malignancy of HN-CICs *in vitro*. (a) SAS-HN-CICs were treated with 0, 10, 25, and 35 *μ*g/mL of YMGKI-1 for 24 hr, afterward, plated onto transwell, and analyzed as Materials and Methods. Dead cells were excluded by trypan blue dye. (b) SAS-HN-CICs were treated with 0, 10, 25, and 35 *μ*g/mL of YMGKI-1 for 24 hr, afterward, plated onto soft agar for 12 day. The colony formation ability of previous cells was examined (see Section 2). Data are means ± SD of triplicate samples from three experiments (***P < 0.005). The same concentration (0.03%) of vehicle (DMSO) was added to all control groups.

**Figure 5:**
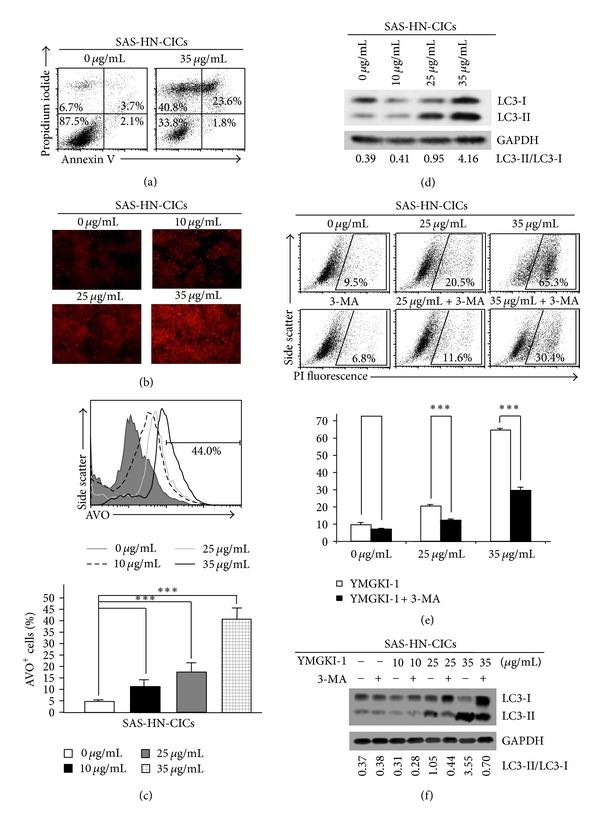
YMGKI-1 treatment induces autophagic cell death in HN-CICs. (a) SAS-HN-CICs treated with 35 *μ*g/mL of YMGKI-1 were costained with Annexin V and Propidium iodide (PI) and examined by flow cytometry. (b) SAS-HN-CICs were treated with YMGKI-1 at different concentration for 24 hr, afterward, stained with acridine orange, and observed under a red filter fluorescence microscope. (c) Acridine orange (AO) positively stained cells of SAS-HN-CICs in (b) were quantitated by flow cytometric analysis (upper panel). The mean fluorescence intensity of AO stained cells was calculated (lower panel). Data are means ± SD of triplicate samples from three experiments (***P < 0.005). (d) Crude cell extract proteins of YMGKI-1-treated SAS-HN-CICs were collected, electrophorized, and further analyzed by immunoblotting against anti-LC3 or anti-GAPDH serum as indicated. (e) SAS-HN-CICs cells were either singly treated with 0, 25, or 35 *μ*g/mL of YMGKI-1, or cotreated with 3-Methylamphetamine (3-MA), an autophagy inhibitor, for 24 hr, afterward, stained with propidium iodide (PI), and then examined by flow cytometry. Data are means ± SD of triplicate samples from three experiments (***P < 0.005). (f) Crude cell extract proteins of SAS-HN-CICs singly treated with YMGKI-1 or cotreated with 3-MA were isolated and analyzed by immunoblotting against anti-LC3 or anti-GAPDH serum as indicated. The immunoactive signal of GAPDH protein of different crude cell extracts was referred as loading control. The same concentration (0.03%) of vehicle (DMSO) was added to all control groups.

**Figure 6:**
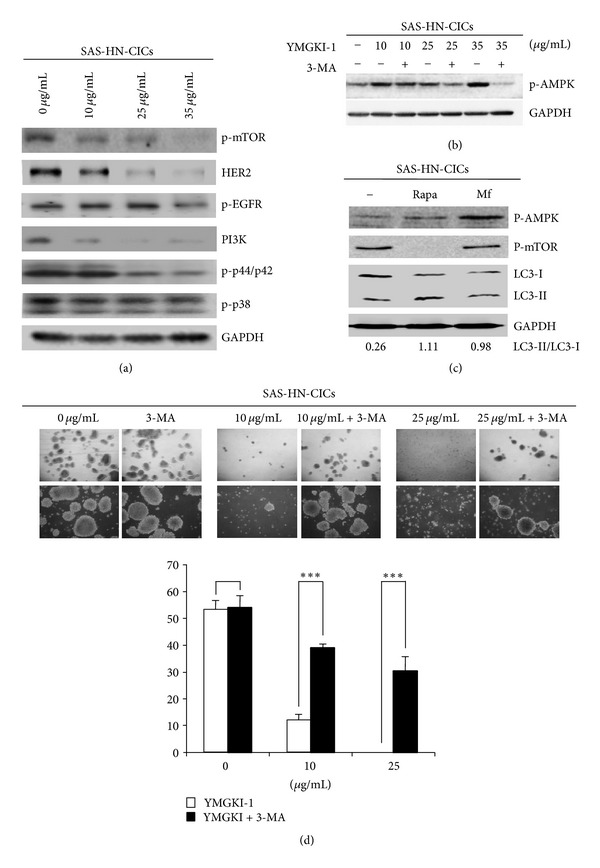
YMGKI-1 treatment dysregulates the autophagy and diminishes the sphere formation ability in HN-CICs. (a) Crude cell extract proteins of YMGKI-1-treated SAS-HN-CICs were collected, electrophorized, and analyzed by immunoblotting against anti-LC3, anti-phosphor-mTOR, anti-HER2, PI3K, anti-phosphor-p44/42 MAPK, anti-phosphor-p38, or anti-GAPDH serum as indicated. (b) Crude cell extract proteins of SAS-HN-CICs singly treated with YMGKI-1 or cotreated with 3-MA were isolated and analyzed by immunoblotting against anti-phosphor AMPK or anti-GAPDH serum as indicated. (c) SAS-HN-CICs were treated with metformin (10 mM) or rapamycin (100 nM) for 96 hr. Consequently, immunoblotting analysis was performed by against anti-phosphor-AMPK, anti-phosphor-mTOR, and anti-LC3 or anti-GAPDH serum as indicated. The immunoactive signal of GAPDH protein of different crude cell extracts was referred as loading control. (d) Single cell suspension of SAS-HN-CICs was treated with YMGKI-1 or cotreated with 3-MA for 24 hr, and the sphere formation ability of YMGKI-1 or cotreated with 3-MA-treated HNCICs cells was examined. Arrows indicated the sphere cells. Data are means ± SD of triplicate samples from three experiments (***P < 0.005). The same concentration (0.03%) of vehicle (DMSO) was added to all control groups.

**Figure 7:**
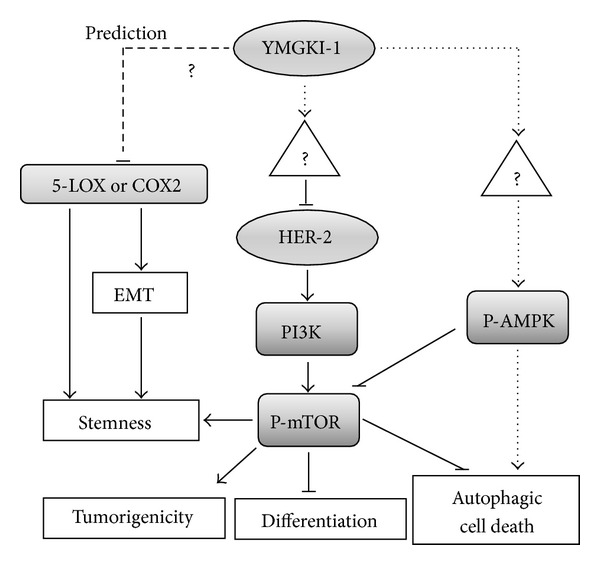
Schematic of the possible signaling pathways regulated by YMGKI-1, through which to inhibit the EMT, stemness properties, and tumorigenicity of head and neck cancer initiating cells (HN-CICs). The HER2/PI3K/MAPK/mTOR signaling pathway plays an important role in self-renewal, survival, and malignancy of CICs. YMGKI-1 would directly or indirectly inhibit the HER2/PI3K/MAPK/mTOR pathway. Further, an YMGKI-1 analogue is predicted to be a potential inhibitor of cyclooxygenase-2 (COX-2) and 5-lipoxygenase (5-LOX) which are related to epithelial-mesenchymal transition (EMT) [7]. YMGKI-1 might also prevent cells from undergoing EMT to gain stem cell properties via inhibiting COX-2 or 5-LOX. (—) refers to known pathway; (- - -) refers to unknown relationship; (……) refers to prediction.

In this study, we utilized the *in vitro* cell-based ALDH activity assay system to screen drugs on targeting cancer initiating cells. YMGKI-1, a purified component of natural product, *Antrodia cinnamomea* mycelia extract, decreased ALDH activity in oral cancer and other cancer cell lines (Figure 1 and Supplementary Table 1). We also showed that YMGKI-1 treatment reduced the stemness properties of parental SAS cells (Figures 1(c) and 1(d)). In addition, we demonstrated that YMGKI-1 suppressed tumor growth in preventive and therapeutic model *in vivo* (Figure 2). Moreover, YMGKI-1 treatment in SAS-HN-CICs abrogated the *in vitro* malignancy and downregulated the stemness properties (Figures 3 and 4). Finally, YMGKI-1 was able to inhibit the growth of HN-CICs by dysregulatory autophagic cell death (Figure 5). Of note, coaddition of YMGKI-1 and autophagy inhibitor (3-MA) to HN-CICs attenuated the cell death, LC3-II accumulation, AMPK activation, and the morphological change of spheroid cells induced by YMGKI-1 treatment (Figure 6). All of these suggest that YMGKI-1 can function as a prospective drug for targeting CICs, and the future research to delineate the function of other YMGKI-1 analogues on HN-CICs will be of benefit for future cancer therapeutics.

Recently, the role of autophagy in the biology of stem cell and cancer initiating cell has been controversial. Cufí and colleagues report that autophagy is positively linked to the maintenance of breast cancer stem-like phenotype [42]. Conversely, others demonstrate that autophagy promotes the differentiation of glioma-initiating cells [17]. Herein, our results indicated that YMGKI-1 can not only induce autophagy but also downregulate the stemness properties and malignancy of HN-CICs (Figures 3 and 5). Although basal autophagic activity in some cells contributes to cytoprotective process against environmental stress [15], our findings suggest that the dysregulatory autophagy induced by YMGKI-1 treatment may effectively contribute to the enhanced cell death in HN-CICs but not in majority HNSCC cells (Figures 3(a) and 5 and Supplementary Figures S1 and S3). Further, we showed that YMGKI-1 induced autophagic cell death in HN-CICs through multiple molecular mechanisms including activation of AMPK and downregulation of PI3K-mTOR pathway (Figure 6). Of note, the aforementioned two signaling pathways center the regulation of autophagy. In addition, there are various signaling cascades that regulate mTOR including the HER2/PI3K (class I) pathway; inhibition of HER2/PI3K (class I) pathway has been shown to induce autophagy in cancer [43].

Under sufficient supply of growth factors and nutrients, the active mTORC1 stimulates growth-related processes such as protein translation, for example, by phosphorylation of S6K1 and 4E-BP, while simultaneously inhibiting self-consuming processes such as autophagy [44]. In opposite, the AMPK activity is enhanced when the intracellular ATP/AMP ratio is decreased. Further, AMPK activation induces autophagy through inhibition of mTORC1 [37]. Therefore, both AMPK and mTOR pathways play the crucial roles simultaneously in YMGKI-1-mediated autophagic HNSCC cell death. To further address the role of AMPK and mTOR on mediating HN-CICs through autophagy, we treated HN-CICs with AMPK activators metformin (Mf) or mTOR inhibitors rapamycin, respectively. As shown in Figure 6(c), HN-CICs treated with metformin or rapamycin displayed induction of autophagy. Nevertheless, we did not observe cell death from HN-CICs under single treatment with either metformin or rapamycin (Supplementary Figure S3B). Because of lack of induced cell death in HN-CICs by single treatment with metformin or rapamycin, it is worth note that YMGKI-1 induces dysregulatory autophagic cell death through multiple mechanisms. It will be of interest to determine how multiple mechanisms regulate autophagic cell death in HN-CICs.

Charafe-Jauffret et al. and Todaro et al. demonstrate that cancer stem cells highly express tumorigenic and metastatic activity [45, 46]. Although cancer stem cells may vary among different types of cancer, YMGKI-1 might have common effect on them. Other than HNSCC, here, we had tested the cytotoxic effect of YMGKI-1 treatment on colon, breast, and lung cancer cell lines. As shown in Supplementary Table 1, the more tumorigenic or metastatic cancer cell lines displayed higher reduction of ALDH^+^ cells under the YMGKI-1 treatment. Together, our data evidences that YMGKI-1 can significantly affect CICs population without discrepancy on different types of tumor.

Conventional anticancer treatments can often transiently shrink tumor volume instead of targeting or killing CICs, which lead to treatment failure, tumor recurrence, and patient death. Cancer initiating cells model suggests that tumors consist of CICs and differentiated cancer cells [3]. In the future, the main treatment strategy will be based on depletion of both the CICs pool and the residual majority cancer cells with combinatorial treatment including CIC-targeted anticancer drugs and conventional cancer therapeutic drugs [47]. Furthermore, in Figure 5(b), we observed that YMGKI-1 effectively inhibited the activation of HER2 but inversely enhanced the expression of phosphor-Src activity (data not shown). The Src kinase activity is closely related to cancerous malignancy [48]. Additionally, Zhang et al. discover that increased Src activation confers considerable Herceptin resistance in breast cancer cells and correlated with Herceptin resistance in breast cancer patients [49]. It is plausible that a combination with YMGKI-1 and Src kinase inhibitor will be a highly effective therapeutic protocol for head and neck cancer treatment. Further research effort with regard to the aforementioned phenomenon is needed in this area.

Together, our present research showed that YMGKI-1, a natural compound from ACME, can specifically and effectively target HN-CICs and diminish the CICs properties and activate dysregulatory autophagic cell death. Therefore, YMGKI-1 treatment might be a potential therapeutic target for HNSCC by eliminating CICs.

## Supplementary Material

Supplementary Table 1. Effect of YMGKI-1 Treatment on Cancer Cell Lines
Supplementary Figure S1. YMGKI-1 affected the cell viability of SAS-HN-CICs (1 × 107 cells/well of 6-well plate) but not parental SAS (5 × 105 cells/well of 6-well plate). A, Cells were treated with 0, 10, 25, 35 and 50 *μ*g/ml of YMGKI-1 for 24 hr, afterward, stained with propidium iodide (PI) and then examined by flow cytometry. B, Morphologies of parental SAS and SAS-HN-CICs with YMGKI-1 treatment.
Supplementary Figure S2. YMGKI-1 induced caspases activation of SAS-HN-CICs. A, Crude cell extract proteins of YMGKI-1 treated SAS-HN-CICs were collected, electrophorized and analyzed by immunoblotting against anti-Cleaved PARP, anti-Cleaved Caspas-3 or anti-GAPDH serum as indicated.
Supplementary Figure S3. HN-CICs treated with metformin or rapamycin displayed slight induction cell death. A, Morphologies of SAS-HN-CICs with metformin or rapamycin treatment for 96 hr. B, SAS-HN-CICs were treated with metformin (10 mM) or rapamycin (100 nM) for 96 hr. , afterward, stained with propidium iodide (PI) and then examined by flow cytometry.
Supplementary Figure S4. SAS xenograft-derived cells were treated with 0, 10, 25 and 50 *μ*g/ml of YMGKI-1 for 24 hr, afterward; the ALDH activity of drug treated cells was examined by flow cytometry. The data are means ± SD of triplicate samples from three experiments (∗∗∗, P < 0.005).
