# Supplementary material for: Active Component of Antrodia cinnamomea Mycelia Targeting Head and Neck Cancer Initiating Cells through Exaggerated Autophagic Cell Death
Source: Evid Based Complement Alternat Med. 2013 Jun 12;2013:946451. doi: 10.1155/2013/946451 (PMC3703405; doi:10.1155/2013/946451)
Supplement: Supplementary file 1 — Supplementary Table 1. Effect of YMGKI-1 Treatment on Cancer Cell Lines Supplementary Figure S1. YMGKI-1 affected the cell viability of SAS-HN-CICs (1 × 10^7^ cells/well of 6-well plate) but not parental SAS (5 × 10^5^ cells/well of 6-well plate). A, Cells were treated with 0, 10, 25, 35 and 50 μg/ml of YMGKI-1 for 24 hr, afterward, stained with propidium iodide (PI) and then examined by flow cytometry. B, Morphologies of parental SAS and SAS-HN-CICs with YMGKI-1 treatment. Supplementary Figure S2. YMGKI-1 induced caspases activation of SAS-HN-CICs. A, Crude cell extract proteins of YMGKI-1 treated SAS-HN-CICs were collected, electrophorized and analyzed by immunoblotting against anti-Cleaved PARP, anti-Cleaved Caspas-3 or anti-GAPDH serum as indicated. Supplementary Figure S3. HN-CICs treated with metformin or rapamycin displayed slight induction cell death. A, Morphologies of SAS-HN-CICs with metformin or rapamycin treatment for 96 hr. B, SAS-HN-CICs were treated with metformin (10 mM) or rapamycin (100 nM) for 96 hr. , afterward, stained with propidium iodide (PI) and then examined by flow cytometry. Supplementary Figure S4. SAS xenograft-derived cells were treated with 0, 10, 25 and 50 μg/ml of YMGKI-1 for 24 hr, afterward; the ALDH activity of drug treated cells was examined by flow cytometry. The data are means ± SD of triplicate samples from three experiments (∗∗∗, P < 0.005). [file 946451.f1.pdf]

Supplementary data

Supplementary Table 1

Effect of YMGKI-1 Treatment on Cancer Cell Lines

Cancer Type (Cell line)	Tumorigenic	Disease	Derived from metastatic site	Dosage (µg/ml)	ALDH (%)	*Percentage reduction of ALDH+ population
Colon						
HCT116	Yes	Carcinoma		0	20.4	0%
				10	11.2	45%
				25	12.8	37%
				35	10.4	49%
HT-29	Yes	Adenocarcinoma		0	22.1	0%
				10	6.5	71%
				25	5.1	77%
				35	4.4	80%
Breast						
T-47D	Yes	Ductal carcinoma	pleural effusion	0	39.8	0%
				10	25.0	37%
				25	14.5	64%
				35	7.83	80%
Lung						
NCI-H520	Yes	Squamous cell carcinoma		0	8.0	0%
				10	4.2	48%
				25	2.6	67%
				35	4.5	44%
NCI-H460	Yes	Carcinoma: large cell lung cancer	pleural effusion	0	13.1	0%
				10	2.0	85%
				25	1.7	87%
				35	1.2	91%
A549	No	Carcinoma		0	7.8	0%
				10	6.0	23%
				25	5.7	27%
				35	4.6	41%

*** Dosage range is from 10 µg/ml to 35 µg/ml.**

Supplementary data

Supplementary Fig. S1

A

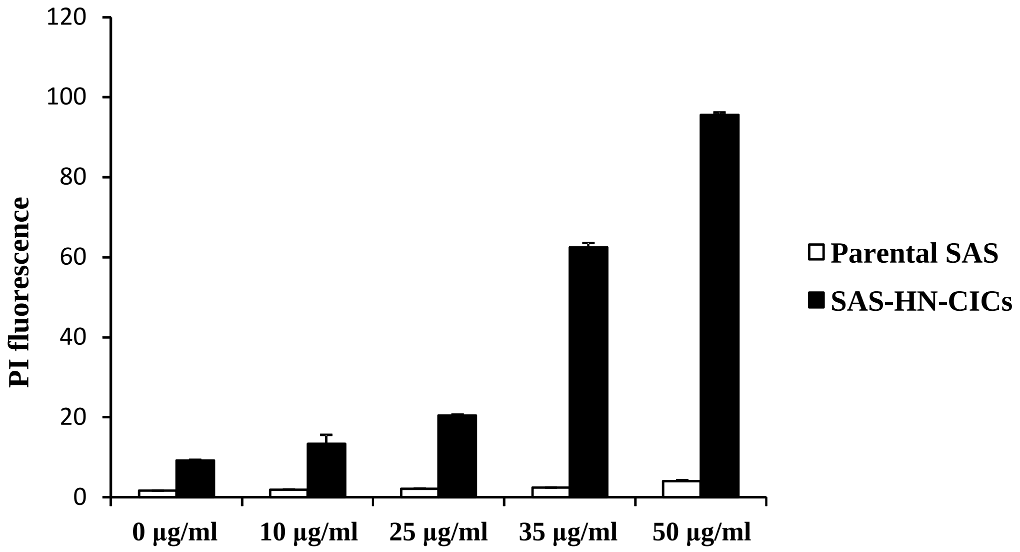

B

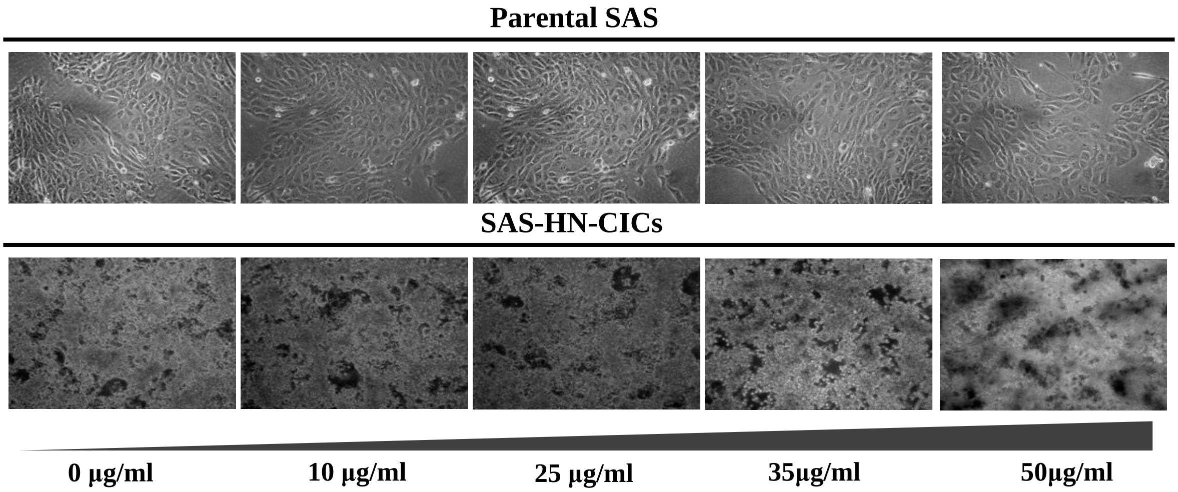

Supplementary Figure S1. YMGKI-1 affected the cell viability of SAS-HN-CICs (1×10^7 cells/well of 6-well plate) but not parental SAS(5×10^5 cells/well of 6-well plate). A, Cells were treated with 0, 10, 25, 35 and 50µg/ml of YMGKI-1 for 24hr, afterward, stained with propidium iodide (PI) and then examined by flow cytometry. B, Morphologies of parental SAS and SAS-HN-CICs with YMGKI-1 treatment.

Supplementary data

Supplementary Fig. S2

A

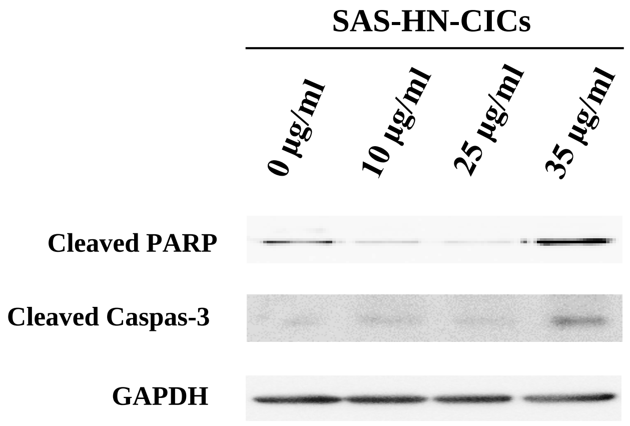

Supplementary Figure S2. YMGKI-1 induced caspases activation of SAS-HN-CICs. A, Crude cell extract proteins of YMGKI-1 treated SAS-HN-CICs were collected, electrophorized and analyzed by immunoblotting against anti-Cleaved PARP, anti-Cleaved Caspas-3 or anti-GAPDH serum as indicated.

Supplementary data

Supplementary Fig. S3

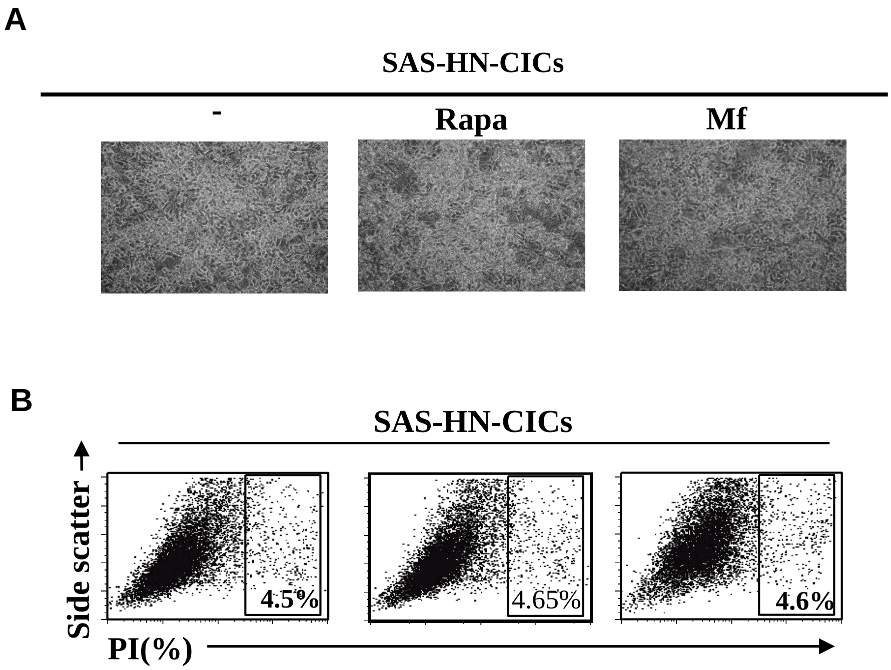

Supplementary Figure S3. HN-CICs treated with metformin or rapamycin displayed slight induction cell death. A, Morphologies of SAS-HN-CICs with metformin or rapamycin treatment for 96hr. B, SAS-HN-CICs were treated with metformin (10 mM) or rapamycin (100 nM) for 96 hr. , afterward, stained with propidium iodide (PI) and then examined by flow cytometry.

Supplementary data

Supplementary Fig. S4

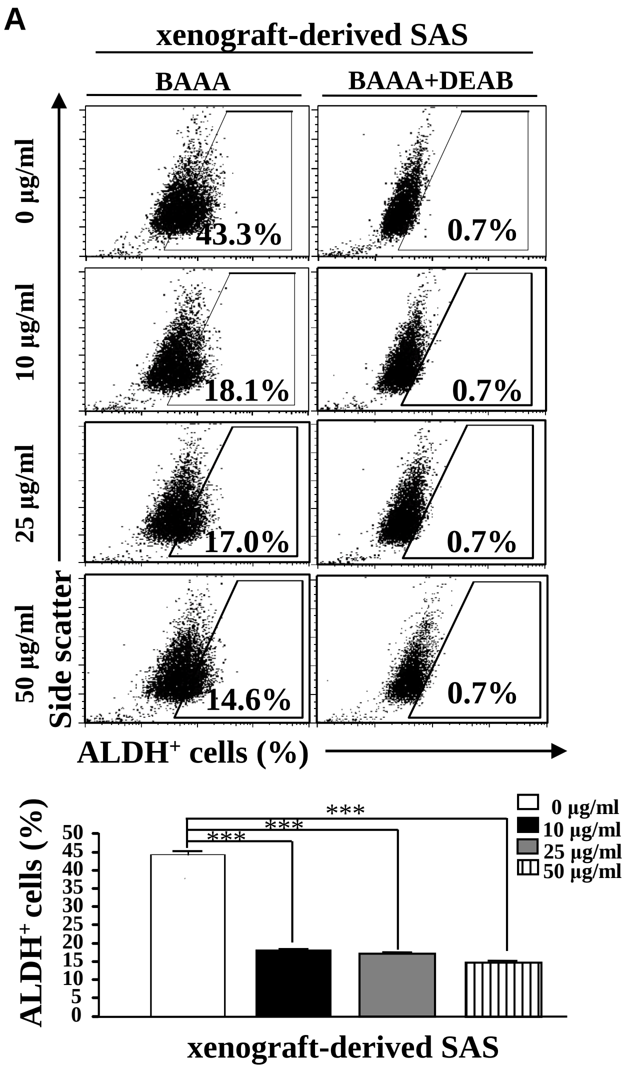

Supplementary Figure S3. SAS xenograft-derived cells were treated with 0, 10, 25 and 50 µg/ml of YMGKI-1 for 24 hr, afterward; the ALDH activity of drug treated cells was examined by flow cytometry. The data are means ± SD of triplicate samples from three experiments (***, $p < 0.005$).

Supplementary data

Supplementary Materials and Methods

Purification of YMGKI-1 from ACME

In brief, the dried and ground mycelium of *Antrodia cinnamomea* was extracted with 95% EtOH. The 95% EtOH extract was then concentrated under reduced pressure. The residues are suspended in H_2O and partitioned with *n*-hexane and then EtOAc. The EtOAc fraction was sequentially chromatographed on silica gel and Sephadex LH-20 columns to obtain YMGKI-1. The structure of YMGKI-1 was elucidated by MS and NMR spectral data.

Protocol to isolate the AFSC cell

In brief, the amniotic fluid-derived stem cells (AFSCs) were harvested from pregnant B6-eGFP mice at 11-13 gestation day. The CD45-Lin- mononuclear cells were isolated using negative selection magnetic bead solution kit. Single-cell suspension of amniotic fluid cells was cultured in IMDM medium supplemented 10% FBS and 100 U/ml penicillin and 100 μg streptomycin (P/S). After 72 hours, non-adherent cells were removed and 10 ng/ml bFGF was added into fresh culture medium. When grown to confluent, adherent cells were detached with trypsin/EDTA and expanded in culture flasks. Stem cell characteristics were confirmed by the expression of phenotype markers, pluripotency associated transcription factors and differentiation capacities. AFSCs between the fifteen and eighteen passage were used in the experiment.
